# Highly Pathogenic Avian Influenza Virus (H5N1) Isolated from Whooper Swans, Japan

**DOI:** 10.3201/eid1409.080655

**Published:** 2008-09

**Authors:** Yuko Uchida, Masaji Mase, Kumiko Yoneda, Atsumu Kimura, Tsuyoshi Obara, Seikou Kumagai, Takehiko Saito, Yu Yamamoto, Kikuyasu Nakamura, Kenji Tsukamoto, Shigeo Yamaguchi

**Affiliations:** National Institute of Animal Health, Kannondai, Japan (Y. Uchida, M. Mase, T. Saito, Y. Yamamoto, K. Nakamura, K. Tsukamoto, S. Yamaguchi); Japan Wildlife Research Center, Taito-ku, Tokyo, Japan (K. Yoneda); Akita Animal Hygiene Service Center of Akita Prefecture, Akita, Japan ( A. Kimura, T. Obara, S. Kumagai)

**Keywords:** Influenza in birds, H5N1 subtype, phylogeny, dispatch

## Abstract

On April 21, 2008, four whooper swans were found dead at Lake Towada, Akita prefecture, Japan. Highly pathogenic avian influenza virus of the H5N1 subtype was isolated from specimens of the affected birds. The hemagglutinin (HA) gene of the isolate belongs to clade 2.3.2 in the HA phylogenetic tree.

Wild birds have been affected by the highly pathogenic avian influenza virus (HPAIV) of H5N1 subtype since 2002. Wild birds are a natural reservoir of type A influenza viruses, which generally cause asymptomatic infection. However, outbreaks of HPAIVs of the H5N1 subtype among residential and migratory birds occurred in the New Territories of Hong Kong Special Administrative Region, People’s Republic of China, in late 2002 (*1*). Furthermore, thousands of migratory birds of several species were affected by HPAIV (H5N1) at Qinghai Lake in western China in 2005 (*2*,*3*). Viruses similar to the ones that caused the outbreak in China eventually spread to Europe (*4*) and Africa (*5*). These events raised concern that migratory birds may play a role in transmission of HPAIVs. The whooper swan (*Cygnus cygnus*) is considered to be a highly susceptible species among wild birds. Infections with HPAIV (H5N1) of this species were reported from China (*6*) and Mongolia in 2005; Iran, Germany (*7*), France, Denmark, the United Kingdom, and Mongolia in 2006; and Russia in 2007. We describe isolation of (H5N1) HPAIV from a whooper swan in Akita prefecture, in the northern part of the main island of Japan.

## The Study

**Figure 1:**
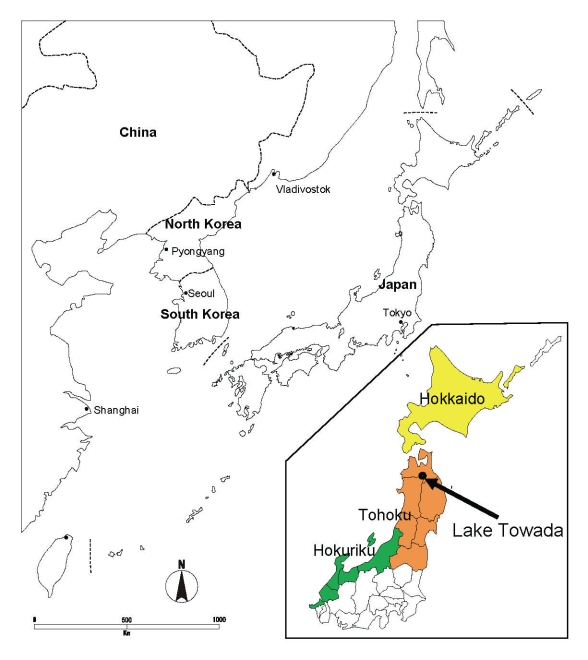
Map of Japan and nearby countries, with enlargement of the northern part of the country (inset) showing location of Lake Towada.

On April 21, 2008, three whooper swans (2 adults and 1 juvenile) were found dead at Lake Towada, Akita Prefecture, Japan (Figure 1). Carcasses were brought into Akita Animal Hygiene Service Center for postmortem examinations. One juvenile swan was also found alive but very weak. It was taken to the Wildlife Protection Center in Akita but had to be euthanized that day in moribund status. Homogenates from the tracheas, cloacas, and internal organs of 3 swans were pooled and inoculated into embryonated chicken eggs for virus isolation.

Hemagglutinating agents from eggs inoculated with each homogenate. Agents were confirmed to be type A influenza viruses by a commercial rapid antigen assay kit and were excluded from being Newcastle disease virus by the hemagglutination inhibition test with Newcastle-specific antiserum. No pathogenic bacteria were isolated. After those tests conducted at the Animal Hygiene Service Center, viruses were brought to the National Institute of Animal Health, Tsukuba, Japan, for further analysis.

The viruses were subtyped as H5N1 with a panel of antiserum, and 1 yielded from cloaca homogenates was designated as A/whooper swan/Akita/1/2008 (WsAk08) and was further analyzed. WsAk08 was shown to be highly pathogenic to chickens by an intravenous administration of 10-fold diluted infectious allantoic fluid. All 8 inoculated chickens died by 26 hours after inoculation. This result coincides with the sequence analysis of the hemagglutinin (HA) gene, showing that the HA protein possesses a series of basic amino acids (PQRERRRKR) at the cleavage site.

**Figure 2:**
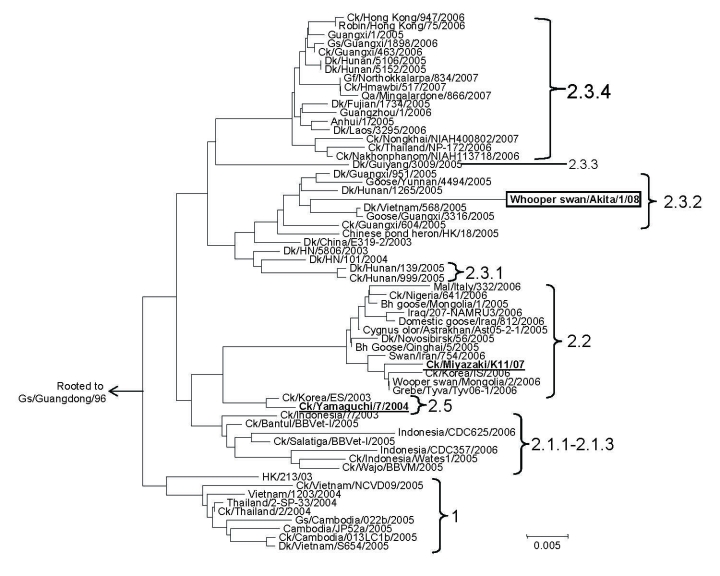
Phylogenetic tree constructed based on the hemagglutinin (HA) 1 region (966 bp) of the HA gene of the highly pathogenic avian influenza viruses (H5N1). Clade designation follows the criteria proposed by the World Health Organization/World Organisation for Animal Health/Food and Agriculture Organization H5N1 Evolution Working Group (*8*). Representative strains of the previous highly pathogenic avian influenza outbreaks in Japan are in **boldface**. Scale bar represents number of nucleotide substitution per site.

Phylogenetic analysis of the HA1 region of the HA gene (Figure 2) showed that WsAk08 belongs to clade 2.3.2 and is clearly distinguishable from the HPAIVs previously isolated in Japan in 2004, A/chicken/Yamaguchi/7/2004 (clade 2.5), and in 2007, A/chicken/Miyazaki/K11/2007 (clade 2.2). Although sequence data were not found in GenBank, A/common magpie/Hong Kong/5052/2007 reportedly resides in the same clade (*9*). Antigenic analysis of WsAki08 with a panel of antiserum and monoclonal antibodies showed low reactivity against antibodies in the panel (Appendix Table). A >32-fold reduction from homologous titers of all hyperimmune serum used was noted with WsAk08. Postinfection duck serum against A/chicken/Yamaguchi/7/2004 and A/chicken/Miyazaki/K11/2007 did not react with WsAk08. None of the monoclonal antibodies against HA protein of A/chicken/Yamaguchi/7/2004 reacted with WsAk08. Thus, WsAK08 is genetically and antigenically distinguishable from the HPAIVs that caused previous outbreaks in Japan.

Sequencing analysis of the entire genome of WsAk08 (GenBank accession nos. AB436731–AB436738) showed that it does not contain amino acid substitutions conferring resistance to adamantane or neuraminidase inhibitors. Unlike many isolates related to Qinghai Lake strains that have spread worldwide, it does not have an E627K substitution in the polymerase basic protein 2. The neuraminidase protein has a 20-aa deletion at aa 49 to 68 in the stalk region. Nonstructural protein 1 has a 5-aa deletion at aa 80 to 84, commonly observed in currently circulating HPAIVs (H5N1) in southeastern Asia.

## Conclusions

Whooper swans breed in northern Eurasia and winter in Europe and eastern Asia i.e., China, the Korean peninsula, and Japan. In Japan, ≈35,000–38,000 whooper swans spend every winter primarily in Hokkaido, Tohoku, and the Hokuriku area (*10*). In the Lake Towada area, ≈300 whooper swans arrive beginning in late October; they leave the area between late March and late April (*11*). In late March, summer birds begin to arrive. According to results of satellite tracking of 8 swans (*12*), as well as the results of banding studies since 1961 (*13*), whooper swans that winter in Japan migrate from the northern end of Honshu Island to eastern Hokkaido, by means of Sakhalin, and reach eastern Siberia, where they breed. To our knowledge, there have been no reports of whooper swans that winter on the Eurasian continent migrating north through Japan.

In light of the migratory route mentioned above, the whooper swans found dead at Lake Towada were most likely recently infected with HPAIV (H5N1) in Japan. It is unlikely that the swans were infected before they flew to Japan in autumn, maintained the virus within the flock, and then suddenly developed the disease after no apparent infections for several months. Although the susceptibility of a certain species of birds to a subtype H5N1 virus may be different depending on the virus strain (*14*), whooper swans as well as mute swans have been considered to be susceptible species to HPAIV (H5N1), as they showed a fulminant course of disease at the outbreak in Germany in 2006 (*7*).

The possibility that the swans were infected by domestic fowl is low because there has been no report of HPAI among domestic fowl in Japan since the beginning of 2008. One possible explanation is that other wild birds brought the virus from outside the country. Although it is not known whether any birds wintering on the continent migrate north through Japan, passage visitor birds such as wader birds migrate from south to north through Japan in spring; summer birds, e.g., egrets, swallows, songbirds, and some raptors, come to Japan from the south in spring for breeding. Also, the possibility of anthropogenic introduction of virus, such as by inappropriate importation of birds, meats, or materials, cannot be excluded.

In conclusion, genetic analysis demonstrates that the virus that killed the 4 swans in Japan in 2008 is genetically distinguishable from the strains that caused previous poultry outbreaks in Japan, ruling out a possibility of resurgence of previously introduced HPAIV in Japan. After the incident we describe, 2 other whooper swan cases of HPAIV (H5N1) infection were confirmed in eastern Hokkaido in early May. Possible involvement of wild birds in the introduction of the virus to Japan requires further scrutiny.

## Supplementary Material

Appendix Table Antigenic analysis of whooper swan/Akita/1/2008 highly pathogenic avian influenza virus (H5N1) versus related isolates*
