# Supplementary material for: Highly Pathogenic Avian Influenza Virus (H5N1) Isolated from Whooper Swans, Japan
Source: Emerg Infect Dis. 2008 Sep;14(9):1427–9. doi: 10.3201/eid1409.080655 (PMC2603097; doi:10.3201/eid1409.080655)
Supplement: Appendix Table — Antigenic analysis of whooper swan/Akita/1/2008 highly pathogenic avian influenza virus (H5N1) versus related isolates* [file 08-0655_appT-s1.pdf]

Appendix Table. Antigenic analysis of whooper swan/Akita/1/2008 highly pathogenic avian influenza virus (H5N1) versus related isolates*

Strain	Chicken hyperimmune serum					Postinfection serum		MAb against Yam704				
	Yam704	Ibaraki105	SA61	Shim58002	Ontario7732	Yam704	MiyaK1107	Y14/3	Y15/2	Y29/3	Y38/3	YY86/1
Whooper swan/Akita/1/2008	160	<20	160	320	160	<10	<10	<10	<10	<10	<10	<100
Chicken/Yamaguchi/7/2004 (Yam704)	**5,120**	1,280	5,120	5,120	2,560	**640**	80	**5,120**	**640**	**640**	**>20,480**	**51,200**
Chicken/Ibaraki/1/2005 (Ibaraki105)	2,560	**5,120**	2,560	5,120	640	10	10	20	<10	80	640	12,800
Chicken/Miyazaki/K11/2007 (MiyaK1107)	1,280	640	2,560	2,560	640	20	**20**	<10	80	<10	10,240	25,600
Tern/South Africa/61 (SA61)	2,560	640	**10,240**	1,0240	2,560	80	20	<10	<10	<10	320	6,400
Swan/Shimane/580/2002 (Shim58002)	2,560	640	5,120	**10,240**	1,280	10	20	<10	<10	<10	640	12,800
Turkey/Ontario/7732/66 (Ontario7732)	640	160	1,280	640	**5,120**	<10	<10	<10	<10	10	80	400

*Postinfection serum specimens were obtained from ducks experimentally infected with Ck/Yamaguchi/7/2004 or Ck/Miyazaki/K11/2007 that survived for 14 d after infection. Monoclonal antibodies (MAbs) were established against Ck/Yamaguchi/7/2004. Homologous titers are in **boldface**.
